# A Systematic Review of Infection Rates and Associated Antibiotic Duration in Acellular Dermal Matrix Breast Reconstruction

**Published:** 2014-11-11

**Authors:** Brett T. Phillips, Muath Bishawi, Alexander B. Dagum, Duc T. Bui, Sami U. Khan

**Affiliations:** ^a^Division of Plastic, Maxillofacial, & Oral Surgery, Duke University Medical Center, Durham, NC; ^b^Division of Cardiovascular and Thoracic Surgery, Duke University Medical Center, Durham, NC; ^c^Division of Plastic and Reconstructive Surgery, Stony Brook University Hospital, Stony Brook University School of Medicine, Stony Brook, NY

**Keywords:** breast reconstruction, acellular dermal matrix, ADM, infection, antibiotics

## Abstract

**Introduction:** Reported infection rates in breast reconstruction with acellular dermal matrix (ADM) can exceed 31%. Prophylactic antibiotics remain controversial due to the absence of evidence-based literature. The purpose of this study was to examine published antibiotic regimens and their associated infection rates in this population. **Methods:** Systematic electronic searches were performed in PubMed, OVID, and the Cochrane databases for studies that reported on prophylactic antibiotic use and infection in patients undergoing ADM breast reconstruction. Two independent authors reviewed studies between 1970 and 2012 for inclusion and data extraction. **Results**: A total of 863 studies were identified and abstracts reviewed. A total of 24 articles were included, with 2148 patients and 3189 ADM reconstructions. Mean infection rates varied between 0% and 31.25%, with a combined average of 11.59%. When comparing antibiotic protocols of less than 24 hours and more than 24 hours, the average infection rate was 2.48% and 13.21%, respectively. **Conclusion:** The current literature lacks consensus on the necessary duration for postoperative antibiotic prophylaxis following breast reconstruction. The potential increased risk of infection associated with ADM remains controversial. Because of the lack of supportive evidence, we do not recommend prolonged postoperative antibiotics in ADM breast reconstruction.

**Level of Evidence:** Therapeutic level III evidence.

In 2013, more than 95,000 breast reconstructions were performed in the United States, with nearly 79% being tissue expander- and/or implant-based surgical procedures.^1^ It has been reported that up to 56% of these cases use acellular dermal matrix (ADM).^2^ In contrast to total submuscular coverage, ADM-assisted breast reconstruction has several reported benefits including additional lower pole implant coverage, allowing the possibility of direct to implant reconstruction, increased tissue expander fill volumes at time of initial operation, and fewer expansions potentially decreasing the time to second-stage reconstruction. These potential benefits have been called into question in the recent literature,^2^^,^^3^ and new guidelines/protocols have been established to better define which patients are ideal candidates.^4^^,^^5^

Although ADM reconstruction may provide significant benefits, extensive controversy exists as to whether ADM itself increases overall complication risks. Multiple systematic reviews and meta-analyses have perpetuated the controversy of whether ADM increases overall complications when used in breast reconstruction.^2^^,^^6^^-^14 Surgical site infection remains a significant complication, with overall breast reconstruction rates ranging from 0% to 29%, with an average of 5.8% in a recent systematic review.^15^

Infection rates in ADM reconstruction have a similar broad range between 0% and 31%.^16^^,^^17^ This is significantly higher than the expected surgical site infection rate of a clean elective operation as defined by the Centers for Disease Control and Prevention.^18^ The surgical placement of an implant under a nonvascularized dermal construct and poorly perfused mastectomy skin flaps has definite potential for increased complication rates. One non–evidence-based approach to this increased complication has been the use of postoperative antibiotics by up to 72% of surgeons.^19^ Prolonged antibiotics have been associated with systemic side effects, drug-resistant bacteria, and *Clostridium difficile* colitis and, in general, should not be used longer than 24 hours postoperatively.^20^ Many antibiotic protocols are described in the plastic surgery literature without a general consensus on terminology or duration, and frequently antibiotics are given longer than the recommended 24 hours. The purpose of this study was to perform a systematic review of the literature, examining the surgical site infection rates and associated antibiotic protocols in ADM-assisted breast reconstruction.

## METHODS

### Search methodology

Electronic searches were performed in PubMed, OVID, and the Cochrane databases for studies that reported on prophylactic antibiotic protocols for patients undergoing breast reconstruction with ADM and available infection rates. MeSH terms used in PubMed are as follows: (“Mammaplasty”[Mesh] AND “Anti-Bacterial Agents”[Mesh]), (“Acellular dermal matrix” AND Breast), (“Acellular dermal matrix” [Mesh] AND Breast), (“Acellular dermal matrix” [Mesh] AND “Anti-Bacterial Agents”[Mesh] AND Breast), (Breast reconstruction AND “Anti-Bacterial Agents”[Mesh]), (“Mammaplasty”[Mesh] AND antibiotics), (“Anti-Bacterial Agents”[Mesh] AND breast reconstruction AND “Infection”[Mesh] AND ADM), (breast reconstruction AND antibiotics), (“Infection”[Mesh] AND breast reconstruction), (“infection AND breast reconstruction). Similar terms were used in OVID. Studies in all languages, including international ones, written from January 1, 1970, to August 2012 were reviewed. References of included articles were also evaluated for further relevant studies for a full circular search.

### Article selection criteria

Prior to data collection, a protocol was drafted and approved by the authors. This included the search criteria, article inclusion and exclusion criteria, and the decision not to contact article authors for further missing information. To be included as part of this review, publications had to report on surgical outcomes of any form of breast reconstruction. All forms of first-stage implant-based breast reconstruction were included (tissue expander and/or implants). Furthermore, articles had to report on the use of ADM in at least one of the patients undergoing reconstruction. The use, or reporting of a specific antibiotic protocol as well as the rate of surgical site infection, was also required for article inclusion. Only retrospective and prospective studies were included; therefore, exclusion criteria included case reports, reviews, editorials, communications, correspondence, discussions, and letters. However, relevant articles that were not necessarily clinical studies were reviewed for relevant references.

Two independent reviewers (B.T.P., M.B.) performed the initial article search and subsequent selection. After duplicate deletion, each article abstract was reviewed for inclusion criteria. If the abstract did not meet clear inclusion or exclusion criteria, the full article was reviewed prior to final categorization.

### Data collection and analysis

Data extraction was performed according to the Cochrane systematic review guidelines. The 2 reviewers extracted data from included articles and placed into an Excel database. Data collection included lead author, publication year, type of study, time range of study patients, type of reconstruction, type of ADM used, timing of reconstruction, total number of patients, total number of reconstructions, antibiotic protocol, and total number of infections. Rates of infection were based on the number of patients included in each study. Studies were further stratified into groups based on antibiotic protocol. Because of poorly defined antibiotic duration protocols, studies were categorized into groups based on the antibiotic regimen described within the article. These included preoperative/intraoperative antibiotics, 5 to 7 days of antibiotics, perioperative and/or antibiotics until drain removal, and nonspecific antibiotic protocol. Additional categorization placed studies into 2 groups: 24 hours or less of antibiotics and more than 24 hours of antibiotics. The number of patients, ADM use, and infections in each group were computed and infection rates were reported. The more than 24 hours of antibiotics group included all original groups besides the preoperative/intraoperative antibiotic studies.

## RESULTS

### Articles reviewed

**Figure 1:**
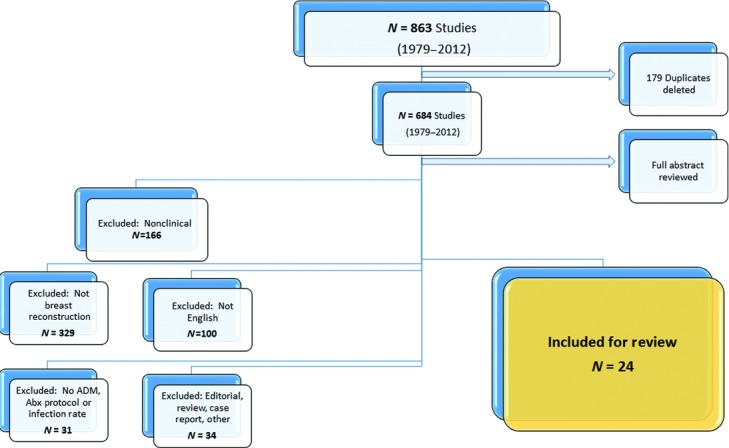
Systematic review flow diagram. ADM indicates acellular dermal matrix; Abx, antibiotics.

Twenty-four articles met our inclusion criteria and were included in this review (Fig 1). Each included article with publication year, study dates, reconstruction type, antibiotic protocol, and infection rates are listed in Table 1.^16^^,^^17^,^21^^-^42 Article demographics are including in Table 2, with the majority of articles being retrospective reviews and only 3 prospective studies. Half of the studies included comparison data for patients undergoing non-ADM reconstructions, whereas the other half reported outcomes only for ADM patients. Multiple ADM types were used, with the majority using AlloDerm (92%) (Lifecell Corp, Branchburg, NJ), and most studies reported results for immediate reconstruction, with only 25% of studies containing patients with delayed reconstruction.

### Antibiotic protocols

Similar to a previously published systematic review,^15^ approximately 7 different protocols were reported. The terminology “prophylactic antibiotics,” “postoperative antibiotics,” and “antibiotics until drain removal” were most commonly used and did not specify an exact antibiotic termination date. These articles encompassed two-thirds of our studies and were placed into a single group. For analysis, all protocols were placed into 1 of 4 groups (Table 3).

### Infection rates

In the 24 included studies, we evaluated 2148 patients with 3189 ADM reconstructions. Using total patients as the denominator for analysis, we found an overall infection rate of 11.59%. When combining the comparative non-ADM patients within the included studies, we had 3357 patients with an overall infection rate that was less than half of the ADM patients at 4.74% (Table 4). When examining infection rates and associated antibiotic protocols, approximately 73% of patients were in the improperly defined perioperative/postoperative antibiotic regimen group, with an overall infection rate of 13.58% (Table 3). The highest infection rate was seen in the “no standard antibiotic protocol” group at 24.39%. This represented only a single study with only 2% of the total patients, although, overall, the highest ADM reconstruction infection was 31.25%.^17^ A different study^41^ was found to have the highest non-ADM reconstruction rate of 39.68%, with a combined ADM and non-ADM infection rate of 35.16% (Table 4).

The overall combined ADM and non-ADM infection rate of 5505 patients was 7.41%. We further stratified our study results to examine the effect of reconstruction timing on infection (Table 5). Rates of infection in ADM patients were increased regardless of whether the study contained immediate or delayed reconstructions. Studies containing patients with delayed ADM reconstruction had similar infection rates to studies with immediate ADM reconstructions (11.29% vs 11.67%). Of interest, patients with delayed non-ADM reconstructions had a higher infection rate than those with immediate non-ADM reconstructions (7.17% vs 4.18%). Unfortunately, because of reporting limitations of the original studies, it was difficult to determine the exact cause for this increase, although delayed reconstruction may have a higher infection rate due to postoperative chemotherapy and/or radiation.

In addition, we condensed our studies into antibiotic protocols of less than or more than 24 hours to evaluate infection rates of studies that followed the Centers for Disease Control and Prevention recommendations (Table 6). A majority of studies (83%) used prolonged duration of antibiotics of more than 24 hours, with an average infection of 13.21%. Studies that used less than 24 hours of antibiotics had one-fifth the infection rate at 2.48%.

## DISCUSSION

The reported complication risks of ADM-associated, implant-based breast reconstructions remain controversial, with studies reporting conflicting complication rates. Four of the 12 comparative studies in our review reported decreased infection rates when using ADM,^26^^,^^27^^,^^37^^,^^41^ whereas the rest still cite increased infections. The average infection rate associated with ADM reconstruction was 11.59% compared with the non-ADM patients at 4.74%. Interestingly, the highest infection rate (39.68%) was discovered in a cohort of non-ADM patients.^41^ We found a wide range of infection rates in patients undergoing breast reconstruction with and without ADM that remain consistent with the recent literature. We also found that definitions of infections varied across all of our studies, and most did not use the standard surgical site infection grading scale. Some studies reported infections using the descriptions “minor” and “major,” which are very subjective and not universally defined. Other studies reported only on infections requiring hospital readmission and/or implant loss. Patients with a diagnosis of cellulitis and treated with oral antibiotics were often excluded. Because of these limitations and the retrospective nature of these articles, it is also likely that the actual infection rates are underreported.

In addition to our review, we examined 10 recent meta-analyses and systematic reviews that provided pooled complication rates and various statistical methods to determine complication rates in patients undergoing ADM breast reconstruction. Adetayo et al^14^ and Newman et al^11^ examined ADM breast reconstruction complications without comparison data and showed infection rates of 9.5% and 5.6%, respectively. Newman et al^11^ reported a 12% overall short-term complication rate associated with ADM. Looking specifically at infection, Jansen and Macadam^12^ found that infection rates ranged from 0% to 11% and noted that a true meta-analysis was difficult due to the lack of validated or standardized outcome measures in the included studies. Three additional meta-analyses compared submuscular coverage versus ADM breast reconstruction and found a 2- to 3-fold increased risk of infection with ADM along with 3- to 4-fold increase in seromas.^8^^,^^9^^,^^13^ Sbitany and Serletti^10^ performed a similar analysis and found a significant increase in seromas but not infections. Combined infection rates in these studies ranged from 4% to 7% in both submuscular and ADM reconstructions, similar to our combined infection rate of 5.13%. These reported infection rates all used the number of reconstructions as the denominator instead of patients. We provided both percentages in our study and believe that studies reporting infection rates should provide both rates to get a better picture of the true infection risk. Our group previously suggested the appropriate unit of analyses when reporting complication rates in a preceding article.^15^ Ho et al^9^ also cited the difficulty in performing combined analysis due to nonuniform definitions of outcome measures. They also recommended the use of a “defined postoperative course of prophylactic antibiotic therapy,” which was infrequently provided or described appropriately.^9^

The most recent and largest reviews have used surgical databases such as National Surgical Quality Improvement Project (NSQIP) and Tracking Operations and Outcomes for Plastic Surgeons (TOPS). Davila et al^7^ reported a 3.8% versus 3.3% infection rate in patients undergoing ADM breast reconstruction and submuscular coverage, respectively. Ibrahim et al^43^ found that superficial surgical site infections were significantly higher in ADM patients at 2.1% than in patients without ADM at 1.6% . Pannucci et al^44^ used the TOPS database and found ADM to be associated with a significant increase in expander or implant loss with an odds ratio of 1.42. These database articles include only short-term complications that are reported up to 30 days. This hardly gives us the true incidence of postoperative complications, especially with respect to infection. According to the Centers for Disease Control and Prevention, implant-based procedures require documentation of surgical site infection up to 1 year after the operation.^18^ One benefit, in contrast to the other meta-analyses in our literature, is that NSQIP data report infection using patient number as the denominator rather than reconstruction.

Since the completion of our review, additional publications in the plastic surgery literature have continued to show increased infection rates in patients undergoing ADM breast reconstruction. Brooke et al^45^ found patients undergoing ADM reconstructions to have infection rates 5 times higher than patients with non-ADM reconstructions with almost 2 times higher overall complications. Liu et al^46^ found a 10.7% infection rate in patients undergoing ADM reconstruction compared with 7.3% in patients undergoing without ADM reconstruction. Although not a primary outcome, McCarthy et al^3^ in a multicenter randomized controlled trial found an infection rate of 8.3% and 3.0% in ADM and submuscular reconstructions, respectively. Overall complication rates were similar between groups. None of these studies reported their respective antibiotic protocols in their methodology. In an effort to decrease overall complication risks, Ganske et al^5^ reported a study with modified postoperative care guidelines to decrease seroma rates. These modifications included postoperative uniform compressive dressings with a surgical bra and removing drains at 20 mL per 24 hours instead of 30 mL per 24 hours. The overall ADM-associated infection rate decreased from 7% to 3.8% following this protocol change. The most significant reduction was observed with major infections, which decreased from 7% to 1.9%. These patients received postoperative antibiotics until drains were removed, with a mean time to removal of 15 days.

In this systematic review, we included 24 articles with at least 7 different antibiotic regimens. The majority of studies were placed into a vague postoperative group. This group had the largest patient size and the second highest average infection rate at 13.58%, with 1 study reporting at 31.25%.^17^ Patients who were reported to obtain more than 24 hours of antibiotics had 5.3 times higher infection rates. In a previous systematic review of almost 15,000 patients undergoing breast reconstruction, we found no difference between patients receiving less than or more than 24 hours of postoperative antibiotics (5.76% vs 5.78%).^15^ Although several limitations can be considered when examining both of these systematic reviews, one can concede that prolonged antibiotics do not appear to decrease infection rates. Higher level evidence is needed to support this continued practice.

Although evidence-based use of appropriate postoperative antibiotics has become a hot topic in the surgical literature, there are few articles addressing this specific issue in breast reconstruction. Clayton et al^47^ compared a prospective cohort of patients receiving only a single preoperative dose of antibiotics with a retrospective group of patients who received antibiotics until drain removal. They found a 4.7-fold increase in surgical site infections, with implant loss in the single preoperative dose group. Their overall reported surgical site infection rate was 27%, with 18% infection rate in the group with antibiotics until drain removal. They concluded that a single dose of antibiotics was not sufficient and that an optimal course of antibiotics is still unknown. Avashia et al^48^ published a retrospective study that examined different antibiotic regimens in patients undergoing ADM breast reconstruction performed by a single surgeon. A series of 12 patients (19 breasts) were given less than 24 hours of perioperative antibiotics and compared with a previous and subsequent cohort of patients who received more than 48 hours of antibiotics. Six of the 12 patients in the less than 24 hours of antibiotics group required implant removal due to infection (6/19 reconstructions). This was significantly higher than the incidence in the other cohorts, 7.9% and 3.2%, respectively. This article claims that administration of 24 hours of antibiotics is clearly not enough for postoperative prophylaxis, basing it upon a comparison of only 12 patients over a 1-month period. Another study by Liu et al,^49^ although examining autologous breast reconstruction, showed no difference in infection rates between patients who received less than or more than 24 hours of antibiotics (19.5% vs 15.5%).

A recent abstract presented by our group provided preliminary results of a randomized controlled trial of patients undergoing ADM breast reconstruction and antibiotic duration.^50^ Patients were randomized to receive 24 hours of perioperative antibiotics versus antibiotics until drain removal. In more than 100 patients, no significant difference was identified in overall infection rates between the 2 groups (15.4% vs 12.2%, respectively). In fact, the 24-hour group had more superficial surgical site infections whereas the latter group had increased implant loss secondary to infection. Final results of this study are subject to a future analysis. It is hoped that this study will be a step toward providing sufficient evidence-based recommendations for postoperative antibiotic prophylaxis in breast reconstruction. Additional large-volume and multicentered trials would assist in answering this question.

There are several limitations of this study that must be considered when interpreting our results. These limitations stem directly from the limitations of the reviewed literature. Specifically, many of the studies suffered from reporting and recall bias, as well as lack of reporting on specifics of their clinical outcomes measures. We excluded studies that specifically looked at infection rates in patients undergoing ADM breast reconstruction because they neglected to mention an antibiotic regimen, which may have impacted our conclusions. Because most of these included studies were retrospective in nature, recall of the antibiotic regimen may not have been completely accurate. Studies stating that preoperative antibiotics were given without mention of postoperative antibiotics might have actually received a postoperative course. In addition, patients were not mutually exclusive and there is a possibility that patients were duplicated in studies. It is common in our field to publish larger series as our sample size increases. As stated previously, the unit of analysis in which our outcomes are reported can significantly change the complication rates. Most articles described infection rates as a function of reconstructions rather than patients. In addition, definitions of infection and antibiotic regimens were not universal across studies. It was also difficult to completely separate patients with immediate and delayed breast reconstruction to accurately assess a difference in outcomes. For the aforementioned reasons, a complete and accurate meta-analysis was impossible. Phillips et al^15^ discussed these inherent literature-reporting drawbacks evident in another systematic review examining a similar question in all forms of breast reconstruction. A uniform breast reconstruction surgical site infection grading scale was provided in addition to recommendations on reporting units of analysis for outcome-based research.

## CONCLUSION

Breast reconstruction is associated with a high infection and overall complication rate. Patients are frequently managed with postoperative antibiotics, although the current literature lacks consensus on the necessary duration following ADM breast reconstruction. The potential increased risk of infection associated with ADM remains controversial, with deficient high-level evidence supporting the necessity for postoperative antibiotics. This study found that ADM reconstruction was associated with a higher infection rate than that reported in patients with non-ADM reconstruction. Interestingly, the lowest rate of infections was seen in patients who received less than 24 hours of antibiotics. Regardless of the type of breast reconstruction, we cannot support the unsubstantiated use of antibiotics past the 24-hour perioperative period in patients with ADM or non-ADM breast reconstruction.

## Figures and Tables

**Table 1:** Systematic review study list

Author (Year)	Study type and date range	Antibiotic protocol	Type of reconstruction	Types of ADM	# Patients	# Infections	% Infections
Breuing and Warren^16^ (2005)	Retrospective review, 2003	Abx until drain removal	IBR (ADM)	AlloDerm	10	0	0.00
Salzberg^21^ (2006)	Retrospective review, 2002–2006	Intraoperative Abx	IBR (ADM)	AlloDerm	49	0	0.00
Breuing and Colwell^22^ (2007)	Retrospective review, 2003–2005	Abx until drain removal	IBR and DBR (ADM)	AlloDerm	29	2	6.90
Ashikari et al^23^ (2008)	Retrospective review, 1988–2007	Preoperative Abx	IBR (ADM)	AlloDerm	65	0	0.00
Spear et al^24^ (2008)	Prospective study, 2004–2005	Abx until drain removal	IBR (ADM)	AlloDerm	43	4	9.30
Topol et al^25^ (2008)	Retrospective review, 2006–2007	Preoperative Abx	IBR (ADM)	AlloDerm/FlexHD	23	2	8.70
Murray et al^26^ (2009)	Retrospective review, 2003–2008	Abx until drain removal	IBR (ADM)	AlloDerm	18	0	0.00
		Abx until drain removal	IBR (non-ADM)	None	121	10	8.26
Nahabedian^27^ (2009)	Retrospective review, 1997–2008	Abx until drain removal	IBR and DBR (ADM)	AlloDerm	76	5	6.58
		Abx until drain removal	IBR and DBR (non-ADM)	None	285	22	7.72
Namnoum^28^ (2009)	Prospective study, 2006–2007	Abx until drain removal	IBR (ADM)	AlloDerm	20	1	5.00
Sbitany et al^29^ (2009)	Retrospective review, 2004–2007	5 d of postoperative Abx	IBR (ADM)	AlloDerm	50	8	16.00
		5 d of postoperative Abx	IBR (non-ADM)	None	50	6	12.00
Antony et al^30^ (2010)	Retrospective review, 2004–2008	Perioperative Abx	IBR (ADM)	AlloDerm	96	11	11.46
		Perioperative Abx	IBR (non-ADM)	None	2025	38	1.88
Chun et al^31^ (2010)	Retrospective review, 2002–2008	Abx until drain removal	IBR (ADM)	AlloDerm	176	24	13.64
		Abx until drain removal	IBR (non-ADM)	None	107	3	2.80
Lanier et al^17^ (2010)	Retrospective review, 2005–2008	Abx until drain removal	IBR and DBR (ADM)	AlloDerm/Strattice/FlexHD	48	15	31.25
		Abx until drain removal	IBR and DBR (non-ADM)	None	71	9	12.68
Nguyen et al^32^ (2010)	Retrospective review, 1998–2008	Nonspecific Abx protocol	IBR and DBR (ADM)	AlloDerm	41	10	24.39
		Nonspecific Abx protocol	IBR and DBR (non-ADM)	None	163	11	6.75
Liu et al^33^ (2011)	Retrospective review, 2004–2009	Abx until drain removal	IBR (ADM)	AlloDerm	192	18	9.38
		Abx until drain removal	IBR (non-ADM)	None	151	5	3.31
Rawlani et al^34^ (2011)	Retrospective review, 2009	Perioperative Abx	IBR (ADM)	FlexHD	84	9	10.71
Cassileth et al^35^ (2012)	Retrospective review, 2005–2010	Abx until drain removal	IBR (ADM)	AlloDerm	43	5	11.63
Chepla et al^36^ (2012)	Retrospective review, 2007–2010	5 d of postoperative Abx	IBR and DBR (ADM)	AlloDerm	145	9	6.21
Endress et al^37^ (2012)	Retrospective review, 2006–2010	7 d of postoperative Abx	IBR (ADM)	Surgimend	28	2	7.14
		7 d of postoperative Abx	IBR (non-ADM)	None	91	9	9.89
Glasberg and Light^38^ (2012)	Retrospective review, 2004–2011	Preoperative Abx	IBR (ADM)	AlloDerm/Strattice	186	6	3.23
Leyngold et al^39^ (2012)	Retrospective review, 2006–2008	Abx until drain removal	IBR and DBR (ADM)	AlloDerm	86	7	8.14
		Abx until drain removal	IBR and DBR (non-ADM)	None	109	3	2.75
Spear et al^40^ (2012)	Retrospective review, 2004–2010	Abx until drain removal	IBR (ADM)	AlloDerm	289	23	7.96
Peled et al^41^ (2012)	Prospective study, 2006–2010	Abx until drain removal	IBR (ADM)	AlloDerm	65	20	30.77
			IBR (non-ADM)	None	63	25	39.68
Weichman et al^42^ (2012)	Retrospective review, 2007–2010	Abx until drain removal	IBR (ADM)	AlloDerm	286	68	23.78
		Abx until drain removal	IBR (non-ADM)	None	121	18	14.88

IBR indicates immediate breast reconstruction; DBR, delayed breast reconstruction; ADM, acellular dermal matrix; Abx, antibiotics.

**Table 2:** Article demographics

Article demographics	*n*	%
Study design		
Randomized controlled trial	0	0.0
Prospective study	3	12.5
Retrospective review	21	87.5
Study type		
ADM only	12	50.0
ADM and non-ADM	12	50.0
Breast reconstruction timing		
Immediate only	18	75.0
Immediate and delayed	6	25.0
ADM type		
AlloDerm	19	79.2
AlloDerm and FlexHD/Strattice	3	12.5
FlexHD	1	4.2
Surgimend	1	4.2

ADM indicates acellular dermal matrix.

**Table 3:** Antibiotic protocol with ADM infection rates

Antibiotic Protocol	# Studies	# Patients	# Infections	Mean infection rate	Infection rate range
Preoperative/intraoperative	4	323	8	2.48%	0%–8.70%
antibiotics					
5–7 days of antibiotics	3	223	19	8.52%	6.21%–16.00%
Perioperative and antibiotics until	16	1561	212	13.58%	0%–31.25%
drain removal					
Nonspecific antibiotic protocol	1	41	10	24.39%	24.39%
Total	24	2148	249	11.59%	0%–31.25%

ADM indicates acellular dermal matrix.

**Table 4:** ADM versus non-ADM comparative infection rates

Type of reconstruction	Studies, (*n*)	Patient, (*n*)	Recon, (*n*)	Infection (P)	Infection (R)	Infection rate (P)	Infection rate (R)	Infection rate range (P)
ADM	24	2148	3189	249	250	11.59%	7.84%	0%–31.25%
Non-ADM	12	3357	4791	159	159	4.74%	3.32%	1.88%–39.68%
Total	24	5505	7980	408	409	7.41%	5.13%	0%–35.16%

ADM indicates acellular dermal matrix; n, number; P, patient; R or Recon, reconstruction.

**Table 5:** Reconstruction timing and infection rates

Study type	# Patients	# Infections	Infection rate
ADM	2148	249	11.59%
Non-ADM	3357	159	4.74%
IBR ADM only	1723	201	11.67%
IBR non-ADM only	2729	114	4.18%
IBR total	4452	315	7.08%
IBR and DBR ADM only	425	48	11.29%
IBR and DBR non-ADM only	628	45	7.17%
IBR and DBR total	1053	93	8.83%
Total	5505	408	7.41%

IBR indicates immediate breast reconstruction; DBR, delayed breast reconstruction; ADM, acellular dermal matrix.

**Table 6:** Antibiotic protocol with ADM infection rates (< or > 24 hours)

Abx protocol	# Studies	# Patients	# Infections	Mean infection rate	Infection rate range
<24 hours	4	323	8	2.48%	0%–8.70%
>24 hours	20	1825	241	13.21%	0%–31.25%
Total	24	2148	249	11.59%	0%–31.25%

Abx indicates antibiotics.
